# Induction of cell cycle arrest at G1 and S phases and cAMP-dependent differentiation in C6 glioma by low concentration of cycloheximide

**DOI:** 10.1186/1471-2407-10-684

**Published:** 2010-12-15

**Authors:** Xijun Liu, Jin-Ming Yang, Samuel S Zhang, Xin-Yuan Liu, David X Liu

**Affiliations:** 1 Department of Neural and Behavioral Sciences, Penn State University College of Medicine, 500 University Drive, Hershey, PA 17033, USA; 2 Department of Pharmacology, Penn State University College of Medicine, 500 University Drive, Hershey, PA 17033, USA; 3 Penn State Hershey Neuroscience Institute, Penn State University College of Medicine, 500 University Drive, Hershey, PA 17033, USA; 4 Penn State Cancer Institute, Penn State University College of Medicine, 500 University Drive, Hershey, PA 17033, USA; 5 Xinyuan Institute of Medicine and Biotechnology, School of Life Science, Zhejiang Sci-Tech University, Hangzhou, Zhejiang 310018, China; 6 Institute of Biochemistry and Cell Biology, Chinese Academy of Sciences, 320 Yue-Yang Road, Shanghai 200031, China

## Abstract

**Background:**

Differentiation therapy has been shown effective in treatment of several types of cancer cells and may prove to be effective in treatment of glioblastoma multiforme, the most common and most aggressive primary brain tumor. Although extensively used as a reagent to inhibit protein synthesis in mammalian cells, whether cycloheximide treatment leads to glioma cell differentiation has not been reported.

**Methods:**

C6 glioma cell was treated with or without cycloheximide at low concentrations (0.5-1 μg/ml) for 1, 2 and 3 days. Cell proliferation rate was assessed by direct cell counting and colony formation assays. Apoptosis was assessed by Hoechst 33258 staining and FACS analysis. Changes in several cell cycle regulators such as Cyclins D1 and E, PCNA and Ki67, and several apoptosis-related regulators such as p53, p-JNK, p-AKT, and PARP were determined by Western blot analysis. C6 glioma differentiation was determined by morphological characterization, immunostaining and Western blot analysis on upregulation of GFAP and o p-STAT3 expression, and upregulation of intracellular cAMP.

**Results:**

Treatment of C6 cell with low concentration of cycloheximide inhibited cell proliferation and depleted cells at both G2 and M phases, suggesting blockade at G1 and S phases. While no cell death was observed, cells underwent profound morphological transformation that indicated cell differentiation. Western blotting and immunostaining analyses further indicated that changes in expression of several cell cycle regulators and the differentiation marker GFAP were accompanied with cycloheximide-induced cell cycle arrest and cell differentiation. Increase in intracellular cAMP, a known promoter for C6 cell differentiation, was found to be elevated and required for cycloheximide-promoted C6 cell differentiation.

**Conclusion:**

Our results suggest that partial inhibition of protein synthesis in C6 glioma by low concentration of cycloheximide induces cell cycle arrest at G1 and M phases and cAMP-dependent cell differentiation.

## Background

Glioblastoma multiforme (GBM) is the most common central nervous system malignancy, whose highly invasive and diffuse nature leaves rare opportunity for cure. Despite aggressive surgical approaches, optimized radiation therapy regimens and the application of cytotoxic chemotherapies, the median survival rates from time of diagnosis range from 12-15 months [1].

Differentiation, as apoptosis, is a defense mechanism by which mammalian cells guard against tumorigenesis. Differentiation therapy, using agents that promote cancer cell differentiation, has been shown to be effective in vitro and in vivo in treatment of several types of cancer cells [2]. Thus, neuroblastoma cells were shown to undergo terminal differentiation upon elevation of intracellular adenosine 3', 5'-cyclic monophosphate (cAMP) concentration after treatment with cyclic nucleotide phosphodiesterase inhibitors or adenylate cyclase activators [3-7]. Cholera toxin was reported to induce malignant glioma cell differentiation via the PKA/CREB pathway [8]. Differentiation of glioma cells can also be achieved by treatment of cells with *Datura stramonium *agglutinin [9]. Notably, *all-trans*-retinoic acid has been used as an agent to induce cell differentiation in clinical treatment of acute promyelocytic leukemia (APL) [10,11], demonstrating the remarkable efficacy of differentiation therapy in treatment of cancers.

Cycloheximide (CHX), originally isolated from *Streptomyces griseus*, has been widely used as an experimental tool to analyze the contribution of protein synthesis to the intracellular signaling and associated cellular function. It inhibits protein translation in mammalian cells at multiple steps, with a particular potency on translation initiation [12]. Protein synthesis consumes enormous energy and resources in cells especially in fast growing cancer cells, and inhibition of protein synthesis by CHX, even in a relatively mild situation, may constitute a major blockade to the cellular functions such as cell proliferation. Indeed, inhibition of synthesis of specific proteins by CHX, such as c-Myc [13], p27 [14] has been interpreted as evidence for the necessity of those cell cycle regulators in either cell proliferation or cell cycle arrest. Similarly, CHX has been shown to either block [15-17] or promote [18-20] gene expression-dependent cell death in different types of cells and experimental paradigms. Surprisingly, a possible biological effect of CHX in cancer therapy, to our knowledge, has never been investigated.

In the present study, we show that low concentration of CHX (LCC) potently inhibited C6 cell proliferation and depleted cells at both G2 and M phases, suggesting blockade at G1 and S phases during which massive protein accumulation is required. Surprisingly, treatment of C6 cell with LCC also induced dramatic morphological transformation that is indicative of cellular differentiation; this change was accompanied by upregulation of differentiation marker glial fibrillary acidic protein (GFAP). In addition, we found that intracellular cAMP levels were dramatically increased in C6 cells treated with LCC, and that this elevated intracellular cAMP is responsible for C6 cell differentiation and upregulation of GFAP.

## Methods

### Cell culture and reagents

Rat C6 glioma cell (ATCC cat. number CCL-107) was cultured in DMEM (Invitrogen) containing 10% FBS (Atlanta Biologicals). Cycloheximide (C4859, CHX), 8-(4-Chlorophenylthio) adenosine 3', 5'-cyclic monophosphate sodium salt (C3912, 8-pCPT-cAMP) and Adenosine (A9251, Ado) were obtained from Sigma. Sources of the antibodies and dilutions are as follows: GFAP (#3670, 1:1000 for both Western and immunostaining), p-Akt (#9271, 1:1000), Cleaved Caspase-3 (Asp175)(1:1000) were from Cell signaling Technology. PARP (P76420, 1:1000) from BD Biosciences. p-JNK (sc-6254, 1:1000), p-Stat3 (sc-7993, 1:1000), Cyclin D1(R-124, 1:1000), Cyclin E (sc-481, 1:750), PCNA (sc-56, 1:500), p-IκB-α (sc-101713, 1:1000), p53 (sc-98, 1:1000), and Ki67 (sc-7846, 1:500) from Santa Cruz Biotechnology. The materials and experimental procedures used in this research were approved by the Biosafety and Recombinant DNA Committee (BRDC) at Penn State University Hershey Medical Center.

### Morphological observations

C6 cells were plated in 6-well plates at a density of 1×10^5 ^per well. Cells were incubated in culture medium containing CHX and/or other reagents at indicated concentrations. Photographs of cells were taken 24 h later.

### Proliferation assay

C6 cells were seeded into 6-well plates at a density of 5×10^5 ^per well and were treated as indicated. Cells were collected after trypsin digestion and counted with a hemocytometer. Values in proliferation charts are expressed as means ± SEM (n = 3).

### Colony formation assay

C6 cells were seeded in 35 mm culture dish at 1,000 cells per dish. After indicated treatment, cells were stained with 0.5% crystal violet solution (made in 25% methanol and stored at room temperature) for 10 min and photographed.

### Flow cytometry

Cell flow cytometry analysis was done as described previously [21]. At the indicated time, cells were trypsinized, washed twice with 1×PBS, and pelleted by low speed centrifugation. Pellet was resuspended with 70% ethanol for 30 min at 4°C. Cells were spun down and were incubated with the DNA-binding dye propidium iodide (PI) solution [0.1% sodium citrate (w/v), 0.1% Triton X-100 (v/v), and 50 mg/L PI in deionized water] for 1 hour at room temperature. Finally, cells were analyzed by a FACS caliber flow cytometer.

### Immunocytochemical staining and confocal microscopy

Experiments were carried out as in [17]. Briefly, cells grown on coverslips were treated as indicated and then fixed in methyl alcohol at -20°C for 10 min. Samples were blocked for 1 h with 5% BSA and then incubated for 1 h at 37°C with the Anti-GFAP antibody. A goat anti-rabbit IgG-TR (Santa Cruz) secondary antibody was used for specific detection of anti-GFAP. Nuclei were stained with Hoechst 33258.

### SDS-PAGE and immunoblotting

Cells in the culture dish were washed with ice-cold PBS and lysed in ice-cold RIPA buffer [50 mM Tris-HCl, pH 7.5, 150 mM NaCl, 0,5% sodium deoxycholate, 0.1% SDS, 1% Nonidet P40]. Cell extracts preparation and Western blotting analysis were done as previously [22].

### Determination of intracellular cAMP level

C6 cells (2 × 10^5^) were seeded in 24-well. After overnight culturing, the cells were treated with CHX, CHX and Ado, at indicated concentrations, and vehicle control for 24 hours. Cells were lysed in 200 μl of 0.1 M HCl and cell extracts were collected after removing cell debris by centrifugation. Quantification of cAMP was carried out using a kit (BioVision) following manufacturer's instruction. Three independent measurements were done for each treatment. cAMP level in untreated C6 cells was arbitrarily set at 1. Data are presented as means ± SEM (n = 3).

## Results

### Potent inhibition of C6 glioma cell proliferation by LCC

**Figure 1:**
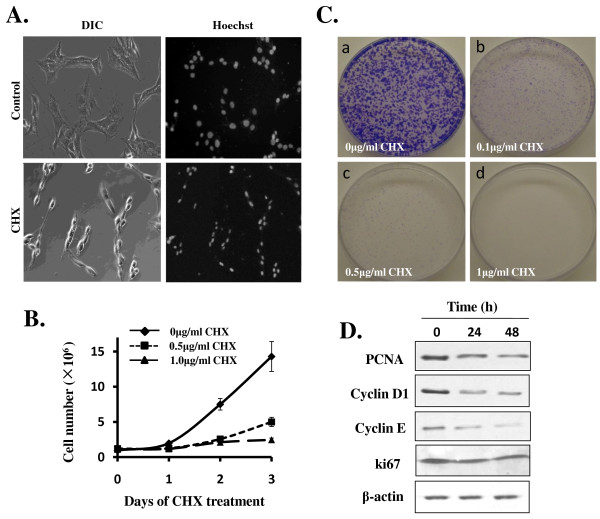
**LCC potently inhibits C6 glioma cell proliferation**. A) C6 cells were seeded in 6-well plates and treated without or with CHX (1 μg/ml) for 48 h. For observation of the integrity of nuclei, cells were stained with Hoechst 33258 and visualized under fluorescence microscope. Both phase contrast (left panels) and fluorescent (right panels) photos were shown (magnification: ×200). B) C6 cells were seeded in 6-well plates at a density of 1.0×10^5 ^per well. CHX were added at concentration of 0 (◆) (as control), 0.5 μg/ml(■), and 1 μg/ml(▲), as indicated. Cells were collected in each of the next 3 days after trypsin treatment and cell numbers were counted using a haemocytometer. Data are presented as means ± SEM (n = 3). C) Colony Formation Efficiency Assay. 1,000 cells were plated in 35-mm tissue culture dishes. After overnight culturing, cells were exposed to different doses of CHX: (a) control; (b) CHX 0.1 μg/ml; (c) CHX 0.5 μg/ml; (d) CHX 1 μg/ml. Photos were taken 6 days later after staining with 0.5% crystal violet. d) Western immunoblotting analysis on cell cycle regulators and indicators. C6 Cells were cultured in 6-well plates and were treated without or with CHX (0.5 μg/ml) for indicated time. Western blotting was performed as described in the Methods.

To see how C6 cell responds to CHX treatment, we performed a dose-response analysis in 6-well plates using CHX concentration ranging from 0 to 100 μg/ml (1 μg/ml = 3.5 μM). High concentration of CHX (50-100 μg/ml) prompted cell death in a few hours (data not shown). LCC (0.1-10 μg/ml) did not cause cell death, permitting us to examine C6 cell response over longer period of time. As shown in Figure 1A, dramatic morphological transformation was observed among the C6 cells after treatment with 1 μg/ml CHX for 24 h; cells were no longer flat, but rather were spindle-shaped with processes (Figure 1A). No cell death was detectable above the background for several days as indicated by Hoechst 33258 staining (data not shown), although the nuclei of the C6 cells treated with CHX were uniformly smaller (Figure 1A). These CHX-treated cells seemed to cease proliferation completely; a cell proliferation assay showed that the normally fast-growing C6 cell no longer proliferated when treated with LCC (Figure 1B). We additionally performed colony-formation assay showing that LCC was extremely potent in inhibiting C6 cell proliferation (Figure 1C). The experiments described in Figure 1B and 1C also demonstrated a dose-dependent response and indicated that the IC_50 _for CHX to inhibit C6 proliferation was less than 0.5 μg/ml.

To see whether cessation of C6 cell proliferation is accompanied with changes in cell proliferation markers and cell cycle regulators in response to LCC, we prepared cell extracts from C6 cells untreated or treated with CHX (0.5 μg/ml) for 24 or 48 h and carried out western blotting analysis. As shown in Figure 1D, cell proliferation markers Ki67 and PCNA and cell proliferation regulators cyclin D1 and cyclin E were all downregulated. Taken together, these data showed that LCC suppresses the expression of several cell cycle markers and stimulators in C6 cell and potently block C6 cell proliferation.

### LCC does not induce apoptosis in C6 cell but depletes cells at G2 and M phases

**Figure 2:**
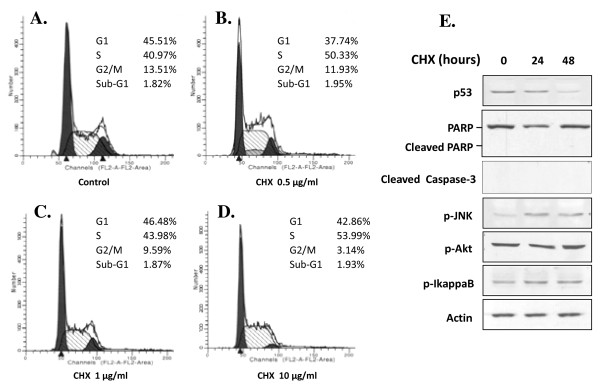
**LCC does not induce apoptosis in C6 cell but depletes cells at G2 and M phases**. A-D) The effect of CHX on cell cycle profile was examined by flow cytometry analysis. C6 cells were seeded in 10 cm dishes at a density of 1.0 × 10^6 ^per dish. After treatment with CHX at indicated concentration for 24 h, cells were trypsinized, washed twice by PBS, and fixed with ice-cold 70% ethanol. DNA content was measured after stained by PI solution [0.1% sodium citrate (w/v), 0.1% Triton X-100 (v/v), 50 mg/ml PI in demonized/distilled water]. Percentages of G2/M (G2) phase cells were indicated. E) Western immunoblotting analysis on molecules that are regulators and indicators of cell stress and cell death. Experiments were done as in Figure 1D.

CHX has been shown to either block [15-17] or induce [18-20] gene expression-dependent cell death in different types of cells and experimental paradigms. In contrast to high concentration of CHX (50-100 μg/ml), which caused death of C6 cells within 6 hours, LCC (0.1-10 μg/ml) did not show any impact on C6 cell survival (Figure 1A); a direct count of the percentage of cell death showed no difference between control and LCC-treated C6 cells (data not shown). We next carried out FACS analysis on C6 cells treated with various LCC. As shown in Figure 2A-D, no sub-G1 population was observed in cells treated with up to 10 μg/ml CHX, further confirming the non-apoptotic effect of LCC on C6 cell. While CHX treatment did not alter the percentage of cells in G1 and S phases, it surprisingly depleted the G2/M population in a dose-dependent manner. We interpret the results to indicate that LCC blocked C6 cell proliferation at both G1 and S phases, which is consistent with the expectation that progression of both G1 and S phases require high level of protein synthesis.

We additionally examined expression of several molecules involved in cell survival and death regulation. As shown in Figure 2E, most of these molecules such as p-Akt and p53 either did not change or underwent changes that in fact favor cell survival. Therefore, we concluded that LCC is not apoptotic to C6 cell, instead it blocks C6 cell cycle progression at the G1 and S phases.

### LCC promotes reversible differentiation of C6 glioma cell

**Figure 3:**
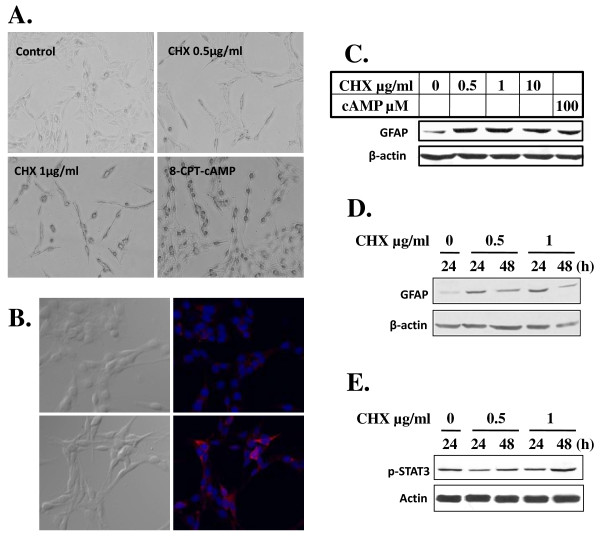
**LCC induces C6 cell differentiation**. A) LCC promotes morphological transformation of C6 cells that indicated cell differentiation. C6 cells were exposed to CHX at indicated concentration and to 8-cpt-cAMP (100 μM) for 24 h. Photos were taken as in Figure 1A (magnification, ×200). B-D) Treatment of C6 cells with LCC upregulates GFAP. In B), C6 cells cultured for 12 h in the absence (upper panels) or presence (lower panels) of CHX (1 μg/ml) were either photographed directly (left panels) or immunostained for GFAP (red) as described in Materials and Methods. Nuclei were stained with Hoechst 33258 as in Figure 1A. In C and D), GFAP expression was assessed in Western immunostaining analysis. Cells were treated with CHX at indicated concentration for 24 h (C) or 24 and 48 h (D). β-actin was used as loading control. E) Western blotting was done as in D) except anti-p-STAT3 was used. β-actin was used as loading control.

The morphological transformation (Figure 1A) and cessation of proliferation (Figure 1B, C) of C6 cells in response to LCC suggested that C6 cells might undergo a process of differentiation. Indeed, as shown in Figure 3A, the smaller and spindle-shaped cells formed after treatment of CHX were remarkably similar to those treated with 8-CPT-cAMP, an analog of cAMP that is known to induce C6 cell differentiation [23,24]. Immunostaining of C6 cells with GFAP, a reliable marker for gliogenesis, indicated a dramatic increase in GFAP production in C6 cells treated with CHX (1 μg/ml) (Figure 3B). This increased expression of GFAP was observed with CHX concentrations ranging from 0.5-10 μg/ml and at comparable levels to 8-CPT-cAMP (cAMP) treatment (Figure 3C), and with highest expression observed at 1 d post treatment (Figure 3D). These data thus showed that C6 cell differentiation induced by LCC is similar to that induced by cAMP. In addition, we demonstrated that the increase of GFAP in C6 cells treated with CHX was accompanied with a measurable increase in p-STAT3 (Figure 2E), which is an obligatory transcription activator for GFAP [25,26].

We further examined whether CHX-induced C6 cell differentiation can be reversed upon CHX withdrawal. We found that differentiated C6 cells reversed their differentiation phenotype and resumed proliferation within 1 d of CHX removal (data not shown). Taken together, the evidence indicated that LCC is able to block cell proliferation and induce reversible differentiation in C6 cell.

### CHX-promoted C6 cell differentiation is associated with an increase in intracellular cAMP and can be blocked by adenosine

**Figure 4:**
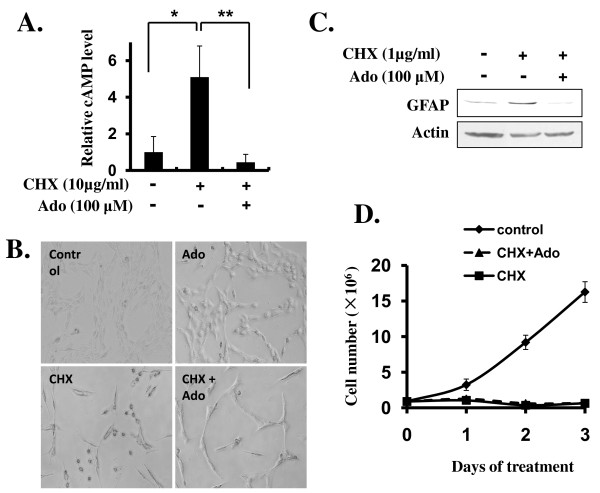
**LCC upregulates intracellular cAMP in C6 cells and this is required for CHX-induced C6 cell differentiation**. A) Treatment of C6 cells with LCC elevated intracellular cAMP and cotreatment with Adenosine (Ado) blocked CHX effect. Experiments were carried out as described in Methods. Data are presented as means ± SEM (n = 3). * *p *< 0.01 (0 vs. 10 μg/ml CHX); ** *p *< 0.001 (10 μg/ml CHX with Ado vs. 10 μg/ml CHX without Ado). B) Adenosine blocked CHX-induced C6 morphological transformation. C6 cells (1.0×10^5 ^per well) were preincubated with or without Ado (100 μM) for 1 h and then cotreated with or without CHX (1 μg/ml) for 24 h as indicated. Phase contrast photos were taken as in Figure 1A. C) Adenosine blocked CHX-induced GFAP upregulation. Western immunostaining was carried out as in Figure 3C except cells were pretreated with or without Ado followed with cotreatment with or without CHX (1 μg/ml) and Ado for 24 h. D) Cell cycle arrest induced by CHX was not affected by Adenosine. Experiments were done as in Figure 1B except cells were pretreated with or without Ado. Data are presented as means ± SEM (n = 3).

Since CHX has been shown to induce increases in intracellular cAMP in several types of cells including neuroblastoma and glioma [27], and cAMP or its analogs are effective inducers of differentiation in C6 glioma cell [23,24](Figure 3A), we hypothesized that LCC promote C6 cell differentiation by its ability to elevate intracellular cAMP in C6 cells. To test this hypothesis, we analyzed the intracellular cAMP in C6 cells untreated or treated with CHX (10 μg/ml). Our data indicated that C6 cell intracellular cAMP concentration increased 5-fold in response to CHX treatment (Figure 4A). To see whether CHX-induced intracellular cAMP increase is responsible for CHX-promoted differentiation transformation, we cotreated C6 cells with CHX and adenosine (Ado) (100 μM), a known inhibitor for cAMP synthesis [28], to reduce intracellular cAMP. As shown in Figure 4A, B, cotreatment of C6 cells with Ado blocked CHX-induced increase in intracellular cAMP in C6 cells (Figure 4A) and prevented CHX-promoted C6 cell phenotypic change of differentiation (Figure 4B). Interestingly, Ado alone did not cause any discernible effect on C6 cell; and it did not block CHX-promoted cessation of C6 cell proliferation (Figure 4B and data not shown). We additionally found that CHX-induced GFAP expression could be effectively blocked by Ado treatment (Figure 4C) and CHX-induced cell proliferation was not reversed with cotreatment of Ado (Figure 4D). Taken together, these data indicated that CHX-induced increase in intracellular cAMP in C6 cells is responsible for CHX-promoted C6 cell differentiation and that the underlining mechanism for CHX-induced cell cycle arrest and that for CHX-induced cell differentiation are independently regulated and separable.

## Discussion

CHX is a potent inhibitor of protein translation in mammalian cells and is widely used in probing the molecular mechanism of various biological processes. While high concentration of CHX (100 μM) was shown to induce apoptosis in hepatocytes both *in vitro *[20] and *in vivo *[19], and we found that high concentration of CHX (50-100 μg/ml, or 180-360 μM) kills C6 cells in a few hours (data not shown), LCC (0.1-10 μg/ml), with an IC_50 _at below 0.5 μg/ml, arrested C6 cell proliferation (Figure 1 and 2) and promoted cell differentiation (Figure 3). Although it was not the focus of this study to identify the critical proteins that are involved in each of the biological processes, the more-than-100-fold disparity in CHX concentrations required for induction of C6 cell death and for C6 cell cycle arrest and differentiation indicated a fundamental difference in the requirement for C6 cell survival, proliferation, and differentiation. We speculated that when C6 cell encounters LCC that shut off the production of a fraction of protein population in the cell, the first to be affected and thus most sensitive to CHX inhibition are the ones that are for cell proliferation. In this situation, C6 cell even increases the production of selective proteins such as GFAP (Figure 3) and c-fos and c-jun [29] that may be needed for differentiation. With increasing concentration of CHX, however, synthesis of proteins whose function involves cell survival, which is more fundamental and strategic to the cell in the long run, will be affected. Thus, the differential response of C6 cell to low and high concentration of CHX may indicate the presence of a priority hierarchy for protein production in C6 cell. Interestingly, we also found that CHX induces U251 human glioma differentiation (data not shown). However, the CHX concentration that is required for U251 to differentiate (10 μg/ml) is about 10 times higher, possibly due to cellular difference in CHX sensitivity or in permeability.

We observed downregulation of a number of cell cycle regulators in C6 cell treated with LCC (Figure 1D), which is generally consistent with the outcome of cell cycle arrest. In addition, we found that p53 is downregulated in C6 cell treated with LCC (Figure 2E). This may explain the fact that apoptosis is not activated in these cells. With regard to differentiation, we saw strong increases in GFAP, a reliable indicator for glial differentiation, both in immunostaining and in Western blotting analyses (Figure 3B, C and 3D). In addition, the increase of GFAP was accompanied with an increase in p-STAT3 (Figure 3E), which is an obligatory transcription activator for GFAP [25,26]. These data convincingly demonstrated that partial blockade of protein synthesis drives forward the differentiation process in C6 glioma cell. Previously, it was shown that C6 cell differentiates when treated with cholera toxin [8] and that other neuroblastomas and glioma also differentiate after treated with cyclic nucleotide phosphodiesterase inhibitors or adenylate cyclase activators [3-7]. These are in line with our findings that LCC upregulated intracellular concentration of cAMP by 5-fold (Figure 4A) and that the increase in intracellular cAMP is required for CHX-induced C6 cell differentiation (Figure 4B, C). These results argue that CHX employs the classical cAMP pathway to inhibit C6 cell proliferation and promote cell differentiation and that intracellular cAMP is a major intermediate that governs glioma cell proliferation and differentiation.

Current therapies used to treat cancer are highly toxic and often nonspecific. Differentiation therapy could be a potentially valuable strategy [2]. In this regard, *all-trans*-retinoic acid has been shown to be remarkable efficient to induce APL cell differentiation in clinical settings [10,11]. Since C6 cell is a well-established cell line for study on tumorigenesis, cancer cell proliferation and differentiation, our data showing that LCC induces C6 cell cycle arrest and cell differentiation will have important impact on the strategy of cancer differentiation therapy.

## Conclusions

Several important conclusions came out from this study. First, unlike high concentration of CHX that provokes C6 cell death in a few hours, LCC is not lethal to C6 cell even after several days of treatment; it causes cell cycle arrest at G1 and S phases during which large amount of proteins, and many of them being critical for cell cycle progression, are synthesized. Second, LCC promotes a profound morphological transformation that indicates C6 cell differentiation, which is further supported by the observation that the expression of glial differentiation marker GFAP is dramatically upregulated. Third, LCC dives forward the C6 cell differentiation process by mobilizing a classical pathway that involves upregulation of intracellular concentration of cAMP. Forth, LCC seems to illicit multiple biological effects in C6 cell, which include cell cycle arrest and cell differentiation, by mechanisms that are clearly distinct and separable. For instance, LCC-promoted C6 cell differentiation but not cell cycle arrest can be reversed by blockade of upregulation of intracellular cAMP; and Ado-induced downregulation of intracellular cAMP, *per se*, does not inhibit C6 cell proliferation. Since CHX is one of the most used reagents in molecular and cellular biology research and C6 cell is a well-established cell line to study cancer cell proliferation, apoptosis, and differentiation, our findings reported here is likely to have a wide implication to future studies in these related fields.

## Competing interests

The authors declare that they have no competing interests.

## Authors' contributions

Conception and design of the project (XL, X-YL, DXL), acquisition, analysis and interpretation of data (XL, DXL), drafting the manuscript (DXL), revision for important intellectual content (J-MY, SSZ, DXL).

## Pre-publication history

The pre-publication history for this paper can be accessed here:

http://www.biomedcentral.com/1471-2407/10/684/prepub
